# Some Practical Suggestions in the Treatment of Diphtheria

**Published:** 1880-09

**Authors:** R. J. Nunn

**Affiliations:** Professor Practice of Medicine in Savannah Medical College. President of Georgia Medical Society, etc.


### The Practitioner.

Vol. I.——*September, 1880.*——No. 9.

ARTICLE I.

### SOME PRACTICAL SUGGESTIONS IN THE TREATMENT OF DIPHTHERIA.

BY R. J. NUNN, M. D., SAVANNAH, GA.

Professor Practice of Medicine in Savannah Medical College. President of Georgia Medical Society, etc.

For many years diphtheria as a local disease was unknown here, indeed its advent seems to have been co-incident with the introduction into the city of an under ground brick sewage system and into the houses of the so called modern improvements, many of which most unsanitary appliances, while of acknowledged convenience, had far better, if health is to be a consideration, be relegated to oblivion, if they cannot be hygienically improved.

Here, then, as in old Edinburgh, there was a locality and there were conditions under which diphtheria did not occur, the report of the Victoria Commission to the contrary notwithstanding.

This condition is however a thing of the past; the present has a far sadder tale to tell.

For some reason at present inexplicable, diphtheria appears by comparison with reports from other places, to have been in this city, a particularly obstinate and remarkably fatal malady.

Every known treatment has been tried over and over again. The thousand and one specifics, which ever and anon flash through our journals, have been eagerly seized upon, faithfully tested and then thrown aside with disgust and disappointment, still bearing with them the same sad history of distressing failure and death. This is about the experience of the whole profession here, whether the cases occured among the rich or the poor, the high or the low, and irrespective of the age or experience of the physician. The cause then, for this heavy percentage mortality of diphtheria, must be sought for in the peculiarities of the locality or the individual idiosyncracies of the people, and treatment must be adopted with a special view to those circumstances which seems to influence the course of the disease.

The report of the treatment of a particular disease, must always be incomplete, unless accompanied with an accurate account of the atmospheric and telluric peculiarities of the locality question, and of the prevailing types of disease there to be found, for, in taking diphtheria as an example it certainly seems, that the treatment with which it is successfully combated in one locality, may be utterly unable to cope with the malady in some other, having different physical surroundings. In no other way can the fact be accounted for, that treatments, although heralded with great flourishes of successes said to be achieved through their means, coming from hilly or mountainous countries or from northern typhoidal latitudes, prove miserable failures when tried in this low flat sandy semi-tropical, malarial country. True, indeed, the hypothesis of Dr. Bellington may be adopted that "Catarrhal Tonsillitis is often mistaken for Diphtheria, and hence the extraordinary successes recorded and credited to various treatments which have as often ignominiously failed." But this would be scarcely creditable to the diagnostic powers of that portion of the profession. (Forty attested cases of Diphtheria, with remarks in diagnosis and treatment by C. E. Billington, M. D., *Medical Record*, March 27, 1880 p. 333.)

During the past year (1879) and until within the last few months an epidemic of diphtheria of moderate exent, but of high percentage mortality prevailed here. It may well be supposed that among other treatments that of local disinfection came in for its full share of trial. First, I believe, brought prominently before the professon by Professor Jacobi in a Paper published in the American Journal of Obstetrics, and lately most ably advocated by Dr. Bellington, (op cit) it has now come to be adopted more or less thoroughly in all the cases I have heard of in this locality, although it has received but a small share of the attention it deserves at the hands of many authors who have written upon the subject.

Towards the close of the epidemic some cases which came under my observation impressed me forcibly with the idea that the local disinfection was in some way at fault ; either the means employed not being sufficiently thorough for the peculiar cases occuring here, or its employment was not commenced at a sufficiently early period of the disease, or perhaps both causes might combine to render the result of the treatment unsatisfactory ; and further, the conviction forced itself upon my mind that the posterior nares and those inaccessible parts of the pharynx adjacent thereto were often the lurking places of the poison, from which it was but imperfectly cleared away or acted upon by the means for local disinfection employed.

The opinion advanced by Dr. Morrill Mackenzie, concerning true nasal diphtheria appears to be confirmatory of this idea.

" True nasal diphtheria," says this experienced authority, "is generally due to the extension of the plastic inflammation from the pharynx." (Diphtheria, its Nature and Treatment, by Morrill Mackenzie, M. D., p. 37.)

While not disputing or discussing the general facts, it seems somewhat a misnomer to consider as a *true* nasal diphtheria, a disease which is stated to be but an extension, presumably by connection, of the pharyngeal affection.

To the practitioner who adopts local disinfection as a treatment, or as a precautionary measure, it makes but little difference where the disease commences, whether in the nasal fossæ, in the pharynx, in the tonsils or elsewhere, he aims to keep all the parts as thoroughly disinfected as he can, malgre all theories as to the point of origin of the membrane.

And so also with the question of the primary lesion. What matters it in a practical point of view, whether it be constitutional or local, provided the fullest precautions are taken on both sides and the patient is not victimized to an unproven and bootless theory?

The difficulties in the way of the settlement of this dispute where each side may at times be right without detracting from the truth of the other, seem so well nigh insurmountable that the point may never be determined with such nicety as to make the distinction of any service.

Diphtheria in its developments seems to be somewhat analogous to those diseases of which vaccine infection may be taken as a type, that is to say, that while it does produce constitutional disturbances, its local manifestation seems to have a predilection for the points of inoculation.

Cases are on record where patients have been inoculated in wounds with the diphtheritic poison from which constitutional disturbances followed, but these disappeared upon the treatment of the local sore. Strange as it may seem while some gentlemen argue from these cases in favor of the constitutional origin of the disease, to me they appear to indicate a constitutional affection as the consequence of the local inoculation.*

The cases referred to seem to have had no throat complications, and hence these cannot be considered a necessary portion of the phenomena of diphtheria.

As the diphtheritic poison is usually inhaled, the materies morbi would be most likely to attach itself to some portion of the respiratory passage, and here its local manifestations would most probably be seen.

In this there appears nothing a whit more extraordinary than that the vaccine scabs should form at the point of inoculation.

If severe local and constitutional symptoms follow a vaccination, a practitioner would scarcely feel justified in wholly neglecting the local, and treating the constitutional disturbances alone, yet this seems to be the position advocated by the ultra believers in the constitutional origin of diphtheria.

In the type of the disease seen here there rarely occurs a case in which there is time to test the question, of the local or constitutional origin of the malady. Blood poisoning comes on so rapidly and puts the patient beyond all treatment or else asthenia so quickly terminates the case, that whatever is to be done must be done at once, and hence local measures should be instituted early, when there is a suspicion of diphtheria, and not wait for a deposit to appear, and the patient vanish about the same time.

It is then in the very inception of the disease that the treatment herein suggested is useful, later on it might by the irritation produced become positively injurious, even if it were possible to carry it out with the restless and resisting patients, such as are most likely to be seen suffering with this affection.

In the cases to which reference has been already made the membrane seemed to descend from the back of the velum palati spreading

* R. M. Griswold, M. D., *Independent Practitioner*, June, 1880 p. 298 and proceedings.

itself over the tonsils, the place of the membrane which was regularly removed, was as regularly supplied from above, extending as before over the tonsillar surface. This then was the stronghold of the disease, the very place most likely to be imperfectly disinfected, or left altogether untouched by any of the treatments heretofore used, and to this point it was determined for the future to devote special attention and with a bent camels hair brush to apply antiseptic solutions freely and thoroughly, until they could be seen issuing from the nostrils, and when possible to use also Thudicum's nasal douche. These measures being of course supplemented by other treatments whenever it should be deemed advisable.

After adopting this course, the number of cases which came under my notice was not large, some ran a very mild course others seemed almost to abort, and none terminated fatally. A like success attended a similar line of treatment applied to cases in other hands. It is quite possible that the severity of the epidemic may about this time have been exhausted, for the disease soon after disappeared.

The nasal douche seems to be a most valuable adjunct to the treatment of diphtheria, and as a means of local disinfection should not be forgotten, it can be used without difficulty even in young children, for this purpose the infant had best be placed face downward with the chest and body resting upon the nurse's lap, the head projecting to one side and the forehead supported by one of the nurse's hands, the nozzle of the douche can then be placed and held in one nostril and the reservoir slowly elevated until the fluid flows through the other.

There is of course no new fact pointed out in bringing more prominently into notice the deposit of the diphtheritic membrane, on the roof of the pharynx and in the posterior nares in certain cases, but it is to the important bearing which such a deposit may have on even ordinary attacks, and the consequent necessity of directing vigorous and early treatment to this locality that I desire to draw special attention.

In one of the best papers published upon this subject for some time.* No mention is made of this mode of treatment except under special circumstances, " when the nares are involved either primarily

* Diagnosis and treatment of diphtheria, by Nathan Jacobson, M. D., Syracuse, N. Y. *Medical Record*, March 20, 1880 p. 309.

or secondarily, in addition to the general measures above mentioned, the local measures applicable to the throat are to be used. Cleanliness, disinfection, a free supply of steam or water with the douche or syringe, are the special points indicated."

This is all very well when the nares are known to be implicated but how is it to be known that they are involved? It is simply absurd to think of practicing rhinoscopy in nine tenths of the cases which come under treatment, indeed it is unfortunately too often a matter of extreme difficulty to introduce a brush; and to manipulate a mirror would be an impossibility, so that either the early implication of the nares must be premised, or special treatment must be deffered until the disease is well advanced as indicated by the nasal discharge.

The following case is illustrative of this position.

A child was attacked with what I was convinced was diphtheria, although no membrane was visible, and as a measure of precaution local disenfection was faithfully employed, but the characteristic membrane soon appeared pushing down from behind the velum, and although it came away upon the straight brush used to paint the tonsils, its place was regularly and rapidly filled from above and this be it observed in spite of the employment of the usual means of disenfection local as well as general. I now determined to supplement this treatment with the use of the bent brush, and Thudicum's nasal douche.

In this particular case the solution applied with the bent brush, was Glycerine, Solution Persulphate of Iron and Carbolic Acid, and with the nasal douche a weak solution of Carbolate of Potass was used. The employment of bent brush was followed by the casting off of a quantity of membrane, the brush was not again used for twenty-four hours, but the douche was frequently employed.

By the time of the next application, the membrane had again just began to show itself, now however only at the upper part of the tonsils coming down from behind the velum as before, and this notwithstanding that the douche and syringe were faithfully employed in the interval. The bent brush was again introduced, this time until the solution appeared at the nostrils, and again its employment was followed as on the previous occasion by a discharge of membrane, after this the deposit on the tonsils was not again renewed, but the use of the bent brush was continued daily until from the disappearance of constitutional symptoms, I felt confident that the disease had subsided.

Another child was attacked shortly afterwards in the same house, I felt convinced it was a case of Diphtheria, yet there was no visilble evidence of it in the throat although there was a slight swelling of the cervical glands. Rhinoscopy was quite impossible but I used the bent brush as before, and soon saw the confirmation of my views by the discharge of a quantity of membrane. The patient made a rapid and most satisfactory recovery.

To medicate the air of the apartments in which these patients were confined, Iodine was employed. This agent is exceedingly manageable, and is known to be an efficient antiseptic. A few crystals thrown upon a saucer and warmed from time to time as the occupants can bear it, is all that is necessary.

It will be observed that this method of applying Iodine differs essentially from that of Dr. Curran which is one of intermittent inhalations.

Some cases which terminated favorably were treated with boracic acid locally and internally. This agent appears to have a most soothing action upon inflamed mucous membranes.

It is soluable in about twenty parts of water which is amply strong for washes and internal administration. Its taste is not disagreeable, in fact is rather alkaline. If desired very strong for local application it can be dissolved in four parts of boiling glycerine from which it does not separate in cooling.

Altogether the results obtained are sufficient to warrant a further trial of this remedy, although I am not prepared to give it a stronger endorsement at present.

The points to which it is desired to give prominence in this paper are:

1st. The necessity for the thorough local disinfection of the roof of the pharynx, the posterior surface of the velum, and the posterior nares when there is the least suspicion of diphtheritic infection, and before the appearance of the membrane.

2nd. The great practical value for this purpose of the bent brush, and Thudicum's nasal douche.

3rd. The use of iodine, as a diffusible disinfectant as being a powerful antiseptic very manageable and well tolerated by the lungs.

4th. To suggest the topical and internal employment of boracic acid.

